# Association of DNA Methylation at *CPT1A* Locus with Metabolic Syndrome in the Genetics of Lipid Lowering Drugs and Diet Network (GOLDN) Study

**DOI:** 10.1371/journal.pone.0145789

**Published:** 2016-01-25

**Authors:** Mithun Das, Jin Sha, Bertha Hidalgo, Stella Aslibekyan, Anh N. Do, Degui Zhi, Dianjianyi Sun, Tao Zhang, Shengxu Li, Wei Chen, Sathanur R. Srinivasan, Hemant K. Tiwari, Devin Absher, Jose M. Ordovas, Gerald S. Berenson, Donna K. Arnett, Marguerite R. Irvin

**Affiliations:** 1 Department of Epidemiology, School of Public Health, University of Alabama at Birmingham, Birmingham, AL, United States of America; 2 Department of Biostatistics, Section on Statistical Genetics, School of Public Health, University of Alabama at Birmingham, Birmingham, AL, United States of America; 3 Department of Epidemiology, School of Public Health and Tropical Medicine, Tulane University, New Orleans, LA, United States of America; 4 HudsonAlpha Institute for Biotechnology, Huntsville, AL, United States of America; 5 Jean Mayer USDA Human Nutrition Research Center on Aging, Tufts University, Boston, MA, United States of America; 6 IMDEA Food, Madrid, Spain; 7 Dean’s Office, College of Public Health, University of Kentucky, Lexington, KY, United States of America; University of Bristol, UNITED KINGDOM

## Abstract

In this study, we conducted an epigenome-wide association study of metabolic syndrome (MetS) among 846 participants of European descent in the Genetics of Lipid Lowering Drugs and Diet Network (GOLDN). DNA was isolated from CD4+ T cells and methylation at ~470,000 cytosine-phosphate-guanine dinucleotide (CpG) pairs was assayed using the Illumina Infinium HumanMethylation450 BeadChip. We modeled the percentage methylation at individual CpGs as a function of MetS using linear mixed models. A Bonferroni-corrected P-value of 1.1 x 10^−7^ was considered significant. Methylation at two CpG sites in *CPT1A* on chromosome 11 was significantly associated with MetS (P for cg00574958 = 2.6x10^-14^ and P for cg17058475 = 1.2x10^-9^). Significant associations were replicated in both European and African ancestry participants of the Bogalusa Heart Study. Our findings suggest that methylation in *CPT1A* is a promising epigenetic marker for MetS risk which could become useful as a treatment target in the future.

## Introduction

Metabolic syndrome (MetS) is a constellation of interrelated risk factors of metabolic origin [1]. Persons with MetS are at twice the risk for cardiovascular disease (CVD) and have a five-fold risk for type 2 diabetes (T2D) [2]. Both genetic and environmental factors play a role in the pathogenesis of MetS. Genetic studies of MetS have often shown that genetic predisposition is attributable to the individual traits rather than the syndrome as a whole and that MetS is a clinical rather than biological phenomenon [3, 4]. However, genome-wide association studies (GWAS) for the individual components of MetS have reported the same loci as being associated with more than one MetS-related trait. To date genetic association studies for MetS have reported modest associations [5].

Few studies have scanned the epigenome for MetS or its constituent risk factors. One study found that individuals with MetS have global DNA hypomethylation relative to those without the syndrome; however, those results were not replicated and that study did not consider methylation at individual loci [6]. Since the epigenome directly impacts gene expression and can be modified by both genetic and environmental factors, epigenetic modifications are prime for further study [7]. We present a DNA methylation epigenome-wide association study (EWAS) of MetS in subjects of European descent from the Genetics of Lipid Lowering Drugs and Diet Network Study (GOLDN). Our top results were validated in the Bogalusa Heart Study (BHS), an external study population comprising participants of both European and African ancestry.

## Research Design and Methods

### Discovery Study

The GOLDN study is composed of families of European descent recruited from field centers in Minneapolis, MN and Salt Lake City, UT. It is part of the NHLBI Family Heart Study and has been described in detail in prior publications [8–10]. Written consent was obtained from each participant during the screening visit; GOLDN included no participants under the age of full legal responsibility (i.e., < 18 yr). The GOLDN study protocol was approved by the Institutional Review Boards at the University of Minnesota, University of Utah, Tufts University/New England Medical Center, and the University of Alabama at Birmingham. We restricted our analysis to individuals aged 30 years and above since many of the risk factors that constitute MetS generally occur in middle age or later. For the present study, 846 individuals aged ≥ 30 years were selected out of 994 participants with available methylation data.

Height, weight, and waist circumference (WC) were measured, and body mass index (BMI) was calculated using standard methods [11]. Blood pressure (BP) was measured with an automated oscillometric device, with participants in a seated position after five minutes of rest. Triglycerides (TG), high density lipoprotein cholesterol (HDLc), and fasting blood glucose (FBG) were measured after ≥ 12 hours of fasting using the Roche/Hitachi 911 automated analyzer. MetS was defined using the National Cholesterol Education Program Adult Treatment Panel III (NCEP-ATP III) guidelines [12].

DNA was isolated from CD4+ T cells harvested from frozen buffy coat samples with positive selection by antigen-specific magnetic beads. The Illumina Infinium HumanMethylation450 BeadChip (Illumina Inc, San Diego, CA) was used to quantify methylation at ~470,000 autosomal cytosine-phosphate-guanine dinucleotide pairs (CpGs) as described in previous publications [8–10, 13]. Principal components (PCs) were generated using the *prcomp* function in R (V 2.12.1) based on the methylation level of all autosomal CpGs that passed quality control. Similarly to previous publications in GOLDN, these PCs were used to adjust for T-cell purity in the association analysis [8–10].

### Statistical Methods

Participant characteristics were compared between individuals with and without MetS by using t-tests. We used a linear mixed model to test for association between methylation at each CpG site and MetS, adjusting for age, sex, study site, and four methylation PCs as fixed effects, and family structure as a random effect using the R kinship package (*lmekin* function). A Bonferroni correction was used to adjust for the number of CpGs tested, where alpha was set to 0.05/470000 = 1.1x10^-7^. Our top CpG finding was tested for association with each individual component of MetS using similar linear mixed models. We also used a t-test to compare methylation levels at our top CpG site by MetS criteria for each component (WC ≥ 102 cm for men and ≥ 88 cm for women, HDLc < 40 mg/dL for men and < 50 mg/dL for women, TG ≥ 150 mg/dL, BP ≥ 130 / ≥ 85 mm Hg, and FBG ≥ 100 mg/dL).

### Replication Population

The Bogalusa Heart Study (BHS), initiated in 1973 as an epidemiologic study of cardiovascular risk factors in children and adolescents, is ongoing [14]. No BHS participants used in this analysis were under the age of full legal responsibility (i.e., < 18 yr), and written consent was obtained from each participant. Study protocols were approved by the Institutional Review Board of the Tulane University Health Sciences Center. Anthropometry, BP, lipids, and glucose were measured after overnight fasting. DNA methylation profiles from whole blood were assessed in 872 individuals who were ≥ 29 years at the time of the methylation assays (603 with European ancestry and 269 with African ancestry). The BHS used the Infinium HumanMethylation450 BeadChip, similarly to GOLDN. To test the association between DNA methylation at CpG sites as the outcome and MetS as a predictor, a linear mixed model was fitted using the R package CpGassoc [15] separately for African Americans and European Americans. Covariates included in the models were age, gender, and white blood cell differential count proportions as fixed effects, and batch as a random effect. The top two CpGs from the GOLDN discovery study were tested for replication in BHS. Therefore, the Bonferroni correction for replication was set at 0.05/2 = 2.50x10^-2^.

The results of the GOLDN and BHS were meta-analyzed by Fisher’s Z score method [16] to obtain the cumulative distribution function of the P-value.

## Results

Characteristics of the GOLDN population (n = 846) with and without MetS are presented in Table 1. The mean age of participants was 53.3 years; 48.5% were female, and 52.7% were recruited from the Minnesota site. By definition participants with MetS in GOLDN (N = 365) had significantly higher BMI, WC, TG, FBG, systolic (SBP) and diastolic (DBP) blood pressure (P<0.0001), and significantly lower HDLc (P<0.0001).

**Table 1:** Descriptive statistics (mean ± standard deviation) of GOLDN and Bogalusa Heart Study participants with and without MetS.

	GOLDN		Bogalusa Heart Study			
	MetS+	MetS-	MetS+*	MetS-*	MetS+^§^	MetS-^§^
N	365	481	249	354	117	152
Age (years)	57.7±12.6	49.9±12.8^†^	44.1±4.1	43.1±4.6**	43.5±4.5	43.1±4.5
Weight (kg)	93.4±16.1	77.3±15.9^†^	100.5±21.7	77.9±17.3^¶^	107.0±24.2	80.8±20.9^¶^
BMI (kg/m2)	32.0±5.1	26.4±4.4^†^	34.3±7.0	27.3±5.3^¶^	37.3±7.8	28.7±7.5^¶^
WC (cm)	107.8±12.6	91.0±13.6^†^	109.4±15.4	91.3±12.7^¶^	111.4±15.0	93.3±15.7^¶^
TG (mg/dl)	199.8±114.1	102.5±54.0^†^	194.5±112.6	106.3±55.3^¶^	143.9±105.2	95.5±67.6^¶^
HDLc (mg/dl)	40.6±10.7	52.3±13.1^†^	39.0±10.1	50.0±14.2^¶^	41.3±10.9	55.6±15.9^¶^
FBG (mg/dl)	110.3±19.4	96.4±11.4^†^	105.0±32.6	87.6±16.0^¶^	113.8±50.4	91.6±31.2^¶^
SBP (mmHg)	124.4±17.8	112.5±15.4^†^	120.1±11.8	111.5±10.7^¶^	133.8±20.5	121.0±17.1^¶^
DBP (mmHg)	71.6±10.4	68.1±8.9^†^	78.2±7.9	71.8±7.2^¶^	84.7±12.3	75.7±10.4^¶^

Data are presented as means ± SD. BMI, body mass index; DBP; diastolic blood pressure; FBG, fasting blood glucose; GOLDN, Genetics of Lipid Lowering Drugs and Diet Network Study; HDLc, high density lipoprotein cholesterol; MetS-, participants without metabolic syndrome; MetS+, participants with metabolic syndrome; SBP, systolic blood pressure; TG, triglycerides; WC, waist circumference.

* Bogalusa Heart Study European Americans;

^§^ Bogalusa Heart Study African Americans;

^†^ P ≤ 0.0001 for MetS+ vs. MetS- comparison;

^¶^ 0.0001 < P ≤ 0.001 for MetS+ vs. MetS- comparison;

** 0.001 < P ≤ 0.01 for MetS+ vs. MetS- comparison.

**Fig 1:**
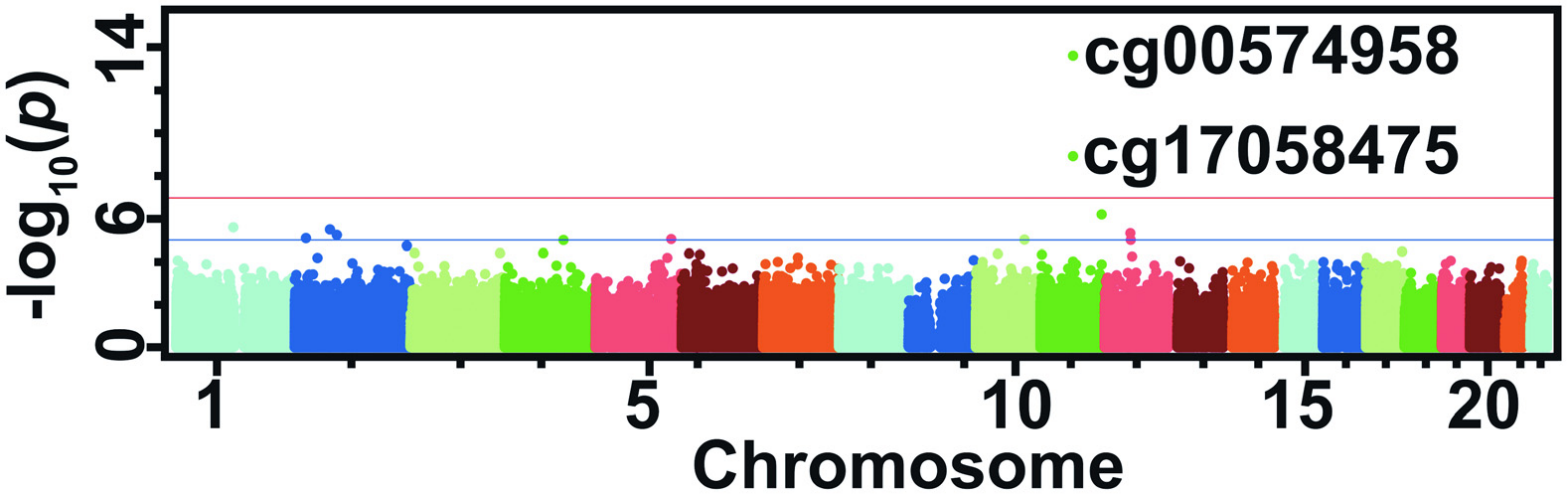
Epigenome-wide association Manhattan plot for MetS in the GOLDN dataset (n = 846). MetS; metabolic syndrome. The blue line indicates a marginal significance level of 1.0 x 10^−5^; the red line indicates the genome-wide significance level of 1.1 x 10^−7^.

**Fig 2:**
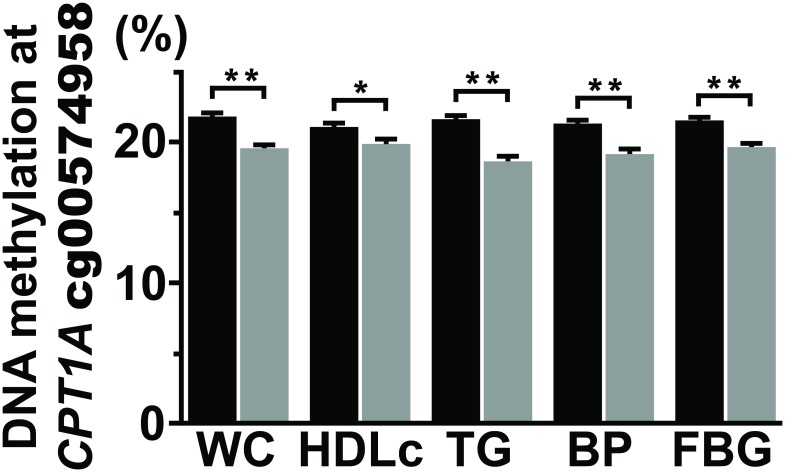
Differences in mean DNA methylation (%) of cg00574958 in *CPT1A* by risk factors of MetS. X-axis shows the criteria met (yes/no) for each component of MetS; Y-axis the mean DNA methylation (%) with error bars showing standard error. *P < 0.01, **P < 0.001. Black bars, MetS criteria not met; gray bars, MetS criteria met. MetS criteria include the following: waist circumference ≥ 102 cm for men and ≥ 88 cm for women; high-density lipoprotein cholesterol < 40 mg/dL for men and < 50 mg/dL for women; triglycerides ≥ 150 mg/dL; blood pressure (systolic/diastolic) ≥ 130 / ≥85 mm Hg; and fasting blood glucose ≥ 100 mg/dL.

The Manhattan plot in Fig 1 shows association results from the GOLDN analysis. Two statistically significant CpGs in the carnitine palmitoyltransferase 1 A gene (*CPT1A*) from the EWAS of MetS in GOLDN are presented in Table 2. Results show lower methylation at cg00574958 (P = 2.6 x 10^−14^) and cg17058475 (P = 1.2 x 10^−9^) is associated with the presence of MetS. The CpGs are located only ~100 base pairs apart, and methylation at each site is significantly correlated (r = 0.85, P < 2.2 x 10^−16^). Fig 2 illustrates the average difference in DNA methylation level (%) with respect to MetS status for each of the five traits that define it. We observed lower methylation at cg00574958 was significantly associated with meeting WC, TG, BP, and FBG criteria for MetS with P < 0.001 and the HDLc criterion with P < 0.01. Upon parallel analyses, results for cg17058475 had the same direction of effect and similar significance for each MetS criteria with the exception that the association result for blood pressure was more marginal, with P < 0.05.

**Table 2:** Association of top two CpGs for MetS in GOLDN discovery and replication in BHS.

CpG	Chromosome	Gene	Location	β (SE)	P
*GOLDN (N = 846)*					
cg00574958	11	*CPT1A*	68607622	-0.026 (3.38x10^-3^)	2.55x10^-14^
cg17058475	11	*CPT1A*	68607737	-0.027 (4.3x10^-3^)	1.21x10^-9^
*BHS Whites (N = 603)*					
cg00574958	11	*CPT1A*	68607622	-0.009 (1.50x10^-3^)	3.96x10^-9^
cg17058475	11	*CPT1A*	68607737	-0.007 (1.89x10^-3^)	2.18x10^-4^
*BHS Blacks (N = 269)*					
cg00574958	11	*CPT1A*	68607622	-0.007 (2.64x10^-3^)	4.90x10^-3^
cg17058475	11	*CPT1A*	68607737	-0.007 (3.30x10^-3^)	1.74x10^-2^

BHS, Bogalusa Heart Study; CpG, cytosine-phosphate-guanine dinucleotide; GOLDN, Genetics of Lipid Lowering Drugs and Diet Network Study.

Characteristics of the BHS European-American and African-American populations are also presented in Table 1. BHS participants are comparable to the GOLDN population except they are, on average, younger (P < 0.0001). The results of the replication analysis for the 2 CpGs in *CPT1A* in the BHS are given in Table 2. Both CpGs were significantly associated with MetS among both European Americans and African Americans. The meta-analysis of all three populations (GOLDN, Bogalusa European Americans and Bogalusa African Americans) demonstrated highly significant P-values for both CpGs—cg00574958 (2.6 x 10^−23^) and cg17058475 (1.4 x 10^−13^). Similarly, the meta-analysis of GOLDN and Bogalusa European Americans also demonstrated significant P-values for both cg00574958 (6.7 x 10^−22^) and cg17058475 (2.06 x 10^−12^).

## Discussion

Our study is the first to report an association between MetS and *CPT1A* DNA methylation. We observed an inverse relationship between methylation at two loci (cg00574958 and cg17058475) in *CPT1A* and MetS overall as well as with specific MetS components. The findings were successfully replicated in BHS participants of European and African Ancestry. The meta-analyses showed highly significant associations of these CpGs with MetS. On balance, our findings indicate that decreased methylation at two intronic loci of *CPT1A* is associated with increased metabolic risk and overall MetS.

Other studies in GOLDN have highlighted this methylation locus for lipids. Specifically, we reported inverse associations between methylation at cg00574958 and cg17058475 and total number and concentration of small LDL particles, large and medium VLDL particles, and LDL diameter [8]. Methylation at the same loci, along with two nearby CpGs, was also found to be inversely related to fasting TG and very low density lipoprotein cholesterol (VLDLc) in a separate analysis, results which successfully replicated in the Framingham Heart Study (FHS) [10]. That report also demonstrated that nearby SNPs from GWAS (located within 1 Mb up or down stream of this locus, representing 1369 SNPs) did not influence these CpGs of interest, and that increased methylation at cg00574958 was associated with decreased *CPT1A* expression [10, 17]. The most significant CpG site (cg00574958) was validated by bisulfite resequencing [10]. Since high TG (≥ 110 mg/dL) is a component of MetS, we tested the association between the other components of MetS (SBP, DBP, FBG, WC, and HDLc as quantitative traits) and cg00574958 while adjusting for TG. Upon adjustment for fasting TG, cg00574958 was associated with WC (P = 7.2 x 10^−6^), HDLc (P = 3.8 x 10^−2^), and FBG (P = 7.6 x 10^−3^) but not with SBP (P = 0.11) or DBP (P = 0.18). These persistent results suggest that *CPT1A* methylation has pleiotropic effects across multiple components of MetS, and the results of this study do not only reflect the gene’s previously demonstrated association with TG.

CPT1A is one of the three isoforms of CPT-1, mostly found in the liver (also known as L-CPT-1). CPT-1 is an important enzyme involved in the regulation of mitochondrial fatty acid oxidation (FAO). It catalyzes the conversion of cytoplasmic long-chain acyl CoA to acylcarnitine (i.e., connecting long-chain fatty acids to carnitine), which then enters into the mitochondria for fatty acid β-oxidation [18]. CPT1A deficiency is a rare metabolic disorder of FAO caused by functional mutations in the gene [19]. GWAS studies have also linked the gene to fatty acid metabolism but not clinical metabolic phenotypes [20, 21]. Several points of evidence suggest that CPT-1 may be a promising target for the development of therapeutic agents against diabetes and obesity; however, a better understanding of metabolic changes following CPT1A manipulation is needed [22]. In particular, a decrease in mitochondrial fatty acid uptake results in elevated intramuscular lipid levels which are associated with insulin resistance. However, CPT-1 inhibition has been linked to reduced FAO, but upregulated glucose oxidation and improved whole-body glucose tolerance and insulin sensitivity in a mouse model [18, 23]. Further supporting the potential clinical relevance of our findings, studies of carnitine supplementation have reported improved FAO, reduced oxidative stress, as well as improved lipid metabolism [24–26]. Overall, our study contributes to the growing body of evidence in support of pursing therapeutics centered on the CPT enzymes and/or their biochemical pathways.

The two CpGs highlighted are located in intron 1 which is an active regulatory region according to ENCODE [27]. This region is a 4 kb promoter-associated region surrounding the transcription start site (TSS) of *CPT1A* in the HepG2 cell line (“Active TSS” according to chromHMM) [28]. There are several transcription factor binding sites near our highlighted CpGs. More than one of the annotated transcription factors is involved in lipid metabolism, including sterol regulatory element-binding proteins (SREBPs), peroxisome proliferator-activated receptor gamma (PPAR-γ), and upstream transcription factor 1 (USF1) [29–31]. The two CpGs are between two CpG islands. Given this functional evidence and observed associations between methylation and gene expression in the previous GOLDN study, we speculate the methylation state of these two CpG sites in intron 1 may be indicative of the promoter activity of *CPT1A*.

Our study has several strengths and a few limitations of note. Among the advantages of our data are the epigenome-wide coverage of methylation variation and successful replication in more than one racial group. Our study is, however, limited by the cross-sectional nature of GOLDN and BHS as well as the focus on DNA derived only from blood cell types. In particular, we observed an inverse association between MetS and methylation at two CpG sites in *CPT1A* (i.e. having MetS is associated with lower methylation). Given that CPT1A promotes fatty acid metabolism (improving lipid levels and glucose) and that prior research in GOLDN and FHS has linked increased methylation at cg00574958 with decreased gene expression, we expected MetS to correlate with higher methylation at that site. Given our findings, we considered that the metabolic environment of MetS may change methylation at this site in favor of gene expression. Additionally, we considered that the relationship between this locus and MetS could differ by tissue type. Notably, data from the Multiple Tissue Human Expression Resource (MuTHER) Cohort shows cg00574958 is positively associated with TG in adipose tissue [32]. Moreover both cg00574958 and cg17058475 are lowly methylated in three of the five cell lines from ENCODE (GM12878/lymphoblastoid cell line, H1-hESC/embryonic stem cells, K562/leukemia cell line), but partially methylated in the HELA-S3/cervical carcinoma and HepG2/hepatocellular carcinoma cell line [27]. Future research is greatly needed to continue to unravel the relationship between CPT1A and MetS as well as its individual components, especially given the potential therapeutic relevance of this finding.

In conclusion, we identified an inverse association between methylation at intronic loci of *CPT1A* and MetS as well as individual MetS components. The significant findings observed in GOLDN replicated in both European-American and African-American populations of BHS, strengthening the validity of our observations and showing similarity across different racial groups. In light of other GOLDN studies highlighting this locus, we confirmed these findings as distinct, suggesting pleiotropic effects. Our study generates the hypothesis that methylation at the two *CPT1A* loci may play a role in an ultimate predisposition towards MetS. Future research could help to determine whether this locus can be treated as a target region for personalized prevention and treatment of metabolic disorders, including MetS and T2D.
